# Twitter and Public Health (Part 1): How Individual Public Health Professionals Use Twitter for Professional Development

**DOI:** 10.2196/publichealth.6795

**Published:** 2017-09-20

**Authors:** Mark Hart, Nichole E Stetten, Sabrina Islam, Katherine Pizarro

**Affiliations:** ^1^ Department of Behavioral Science and Community Health University of Florida Gainesville, FL United States

**Keywords:** Twitter, social media, public health, technology transfer, blogging

## Abstract

**Background:**

The use of social networking sites is increasingly being adopted in public health, in part, because of the barriers to funding and reduced resources. Public health professionals are using social media platforms, specifically Twitter, as a way to facilitate professional development.

**Objective:**

The objective of this study was to identify public health professionals using Twitter and to analyze how they use this platform to enhance their formal and informal professional development within the context of public health.

**Methods:**

Keyword searches were conducted to identify and invite potential participants to complete a survey related to their use of Twitter for public health and professional experiences. Data regarding demographic attributes, Twitter usage, and qualitative information were obtained through an anonymous Web-based survey. Open-response survey questions were analyzed using the constant comparison method.

**Results:**

“Using Twitter makes it easier to expand my networking opportunities” and “I find Twitter useful for professional development” scored highest, with a mean score of 4.57 (standard deviation [SD] 0.74) and 4.43 (SD 0.76) on a 5-point Likert scale. Analysis of the qualitative data shows the emergence of the following themes for why public health professionals mostly use Twitter: (1) geography, (2) continuing education, (3) professional gain, and (4) communication.

**Conclusions:**

For public health professionals in this study, Twitter is a platform best used for their networking and professional development. Furthermore, the use of Twitter allows public health professionals to overcome a series of barriers and enhances opportunities for growth.

## Introduction

Facebook, YouTube, Instagram, and Twitter are well-known social networking sites (SNS) that 69% of adults use daily in the United States [1,2]. On a global scale, as of January 2017, over 2.7 billion people, or 37% of the planet’s population, are characterized as active social media users [1]. As the use of SNS has grown in popularity, its applications have expanded beyond personal communication [3]. In particular, one field is increasing its use of social media specifically for professional development is public health.

The practice and discipline of public health focuses on a broad range of tasks to accomplish its ultimate goal of disease and injury prevention among an entire community or population [4]. These preventative measures derive from 10 essential public health services listed by the Centers for Disease Control and Prevention [4]. The final essential public health service listed states that research is key to providing “new insights and innovative solutions to health problems” [4]. This service is completed through collaboration among public health researchers and public health workers and through sufficient funding [5]. Unfortunately, public health funding is continually being cut from health departments and from research purposes, which in turn significantly reduces and eliminates funding for collaboration and networking among public health professionals [5,6].

To bypass the barriers of funding, public health professionals have turned to technology and social media for professional development, using sites such as LinkedIn and Twitter [7,8]. Most SNS share common features, including the ability of users to create public profiles within websites that connect people through common interests and personal relationships, which exponentially grow as more information is provided. Whereas many of the sites have similar features, users often stratify the sites based on different usage patterns; for example, Facebook is often used for friends and family, and LinkedIn is used for employment purposes [8]. Twitter, with 313 million active users, is unique in its function of communicating in 140 characters or less and the use of the hashtags, thus allowing public health professionals to connect and communicate quickly and efficiently [9]. Twitter is used worldwide, allowing communication through numerous languages and can connect users of vastly different geographical and socioeconomic areas. The public space Twitter provides also allows public health workers to network within their specialties, ultimately allowing for better understanding and initiation of the 10 public health essential services.

This use of Twitter, connected with the theoretical framework of social media functionality, shows professional development functions specifically in the constructs of conversation and groups, by not only providing networking for future employment opportunities but also a pool of professional associates fostering learning experiences [10]. During conferences and other important public health meetings, public health organizations are tweeting updates while attending as well as reporting on current research being conducted [7]. Public health organizations are also using Twitter as a form of journal club by choosing an paper and then letting public health professionals tweet discussions and questions with other professionals in real time [8]. Whereas research has explored how public health organizations have adjusted to a financial crisis by overcoming barriers through social media, research has yet to explore how individual public health professionals use social media, specifically Twitter, to enhance their formal and informal professional development [8].

## Methods

### Participants

Using Twitter’s keyword search, the phrases “public health practitioner,” “MPH” (Master’s in Public Health), “public health,” and “APHA” (American Public Health Association) were used to identify potential participants. The researchers sifted through the results to achieve the sample of 200 public health–affiliated Twitter users. Inclusion criteria comprised Twitter users with active accounts and minimums for number of tweets and followers (over 150 followers). Active accounts were defined as users who had posted content on their Twitter page in the past 3 weeks. Additionally, omitted from the sample were users who primarily were not engaged in general public health topics, public health organizations, and those who were identified as working solely in academia. Practicing public health professionals were the target population. The 200 public health professionals identified were asked through a direct Twitter message whether they would participate in a survey asking questions on their Twitter usage related to public health and their professional experiences. Those responding affirmatively were sent a direct link for our survey housed on an encrypted direct server, which was available for 1 month; ultimately, 49 people participated.

### Materials

The first 5 survey items asked for demographic characteristics and the following 5 items specifically asked about Twitter usage. A modified version of an existing scale assessing perceived usefulness and ease of use regarding technology was applied [11]. The following questions asked participants to rate the following questions on a 1 (strongly disagree) to 5 (strongly agree) Likert scale: (1) using Twitter improves my job performance; (2) using Twitter increases my productivity; (3) using Twitter enhances my effectiveness on the job; (4) using Twitter makes it easier to expand my networking opportunities; and (5) I find Twitter useful for professional development. The last 3 questions were open-response questions providing a participant the opportunity to not only address personal meaningful practices but also gather insight on how Twitter has been used to advance professional development. Open-ended questions specifically asked included the following: (1) How has Twitter affected your standing within the field of public health? (2) How has Twitter affected your work in the field of public health? and (3) What advice would you give to other public health professionals considering the use of Twitter?

### Procedure

Through the use of a college departmental Twitter account, specifically set up for this study, a single tweet briefly linked individuals to the electronic questionnaire on Qualtrics (Qualtrics, Provo, UT, USA). Interested participants were routed to a consent webpage before proceeding with the survey. All responses were confidential, and researchers were blind to which public health professionals responded from the 200 participants who were sent the initial direct message. Respondents did not receive any compensation for their participation. All research was approved by the institutional review board of the University of Florida.

### Data Analyses

Frequency counts were used to determine the demographics of the survey respondents (Table 1). Mean, variance, and standard deviation were used to calculate the usefulness and ease of Twitter (Table 2). All quantitative data were analyzed using Statistical Package for the Social Sciences (SPSS). The constant comparison method was used to analyze the open-response survey questions. This method is specifically used to reduce the text (data) into manageable units and, in turn, coded information [12-14]. The process began with 2 trained researchers (SI and NS) open-coding the responses to discover themes and subthemes [12-14]. After open-coding, specific themes were chosen, with both researchers (SI and NS) separately coding the responses by themes [12-14]. After the final coding, the 2 researchers checked to ensure that the responses matched, and discrepancies were decided by a third party who was also a trained researcher in the constant comparison method (MH) [12-14].

## Results

### Participants

Overall, 49 participants responded and completed the survey (response varied by question). Of those, 51% were females (n=25), 47% were males (n=23), and 2% did not identify with a gender (n=1). The majority of participants were aged between 25 and 34 years (45%, n=22). The second majority of participants were aged between 35 and 44 years (24%, n=12; Table 1). Whereas 59% (n=27) of the participants were located in the United States, 41% (n=19) were located outside the United States. The majority of the participants have worked in the field of public health for more than 10 years (43%, n=21), followed by 5 to 9 years (20%, n=10), and 0 to 4 years (37%, n=18). The majority of participants had obtained their master’s degree (52%, n=25; Table 1).

### Usefulness and Ease

The scale asked 5 questions about the usefulness and ease of Twitter on a 5-point Likert scale. “Using Twitter improves my job performance” has a mean of 3.80 (standard deviation [SD] 0.82, n=49), “Using Twitter increased my productivity” has a mean of 3.35 (SD 0.86, n=49), “Using Twitter enhances my effectiveness on the job” has a mean of 3.80 (SD 0.89, n=49), “Using Twitter makes it easier to expand to my networking opportunities” has a mean of 4.57 (SD 0.74, n=49), and “I find Twitter useful for professional development” has a mean of 4.43 (SD 0.76, n=49; Table 2, modified from Davis [11]). Overall, results show that public health professionals find Twitter the most useful and easiest to use for networking and professional development over job performance, productivity, and effectiveness.

**Table 1:** Demographics of survey respondents.

Characteristics		Frequency, n (%)
**Gender (n=49)**		
	Female	25 (51)
	Male	23 (47)
	Unspecified	1 (2)
**Age in years (n=49)**		
	18-24	2 (4)
	25-34	22 (45)
	35-44	12 (24)
	45-54	8 (16)
	55-64	5 (10)
**Location (n=46)**		
	United States	27 (59)
	Outside United States	19 (41)
**Years worked in public health (n=49)**		
	0-4	18 (37)
	5-9	10 (20)
	More than 10	21 (43)
**Education level (n=48)**		
	Some college	1 (2)
	Bachelor’s degree	8 (17)
	Master’s degree	25 (52)
	Professional degree	5 (10)
	Doctorate degree	9 (19)

**Table 2:** Usefulness and ease of use of Twitter on a 5-point Likert scale.

Survey questions	Mean	Variance	Standard deviation	Total responses, n
Using Twitter improves my job performance	3.80	0.67	0.82	49
Using Twitter increased my productivity	3.35	0.73	0.86	49
Using Twitter enhances my effectiveness on the job	3.80	0.79	0.89	49
Using Twitter makes it easier to expand my networking opportunities	4.57	0.54	0.74	49
I find Twitter useful for professional development	4.43	0.58	0.76	49

### Open-Response Questions

Qualitative inquires comprised the last 5 items on the electronic survey. Common across sections emerged from the data, resulting in 4 major themes: (1) geography, (2) continuing education, (3) professional gain, and (4) communication.

#### Geography

A significant number of free responses emphasized the absence of locational factors that would otherwise present limitations in meeting other professionals and colleagues, as is evident from the following responses:

Twitter has enabled me to connect to colleagues across all professions and geography. That would not have been possible without the use of Twitter.

Respondent #8

I have had conversations with people all across the world who I would normally have no opportunity to meet. This has been everyone from prominent professors, presidents of royal colleges, journalists, to patient advocates and medical students. Notably, I have met quite a few of these people at conferences, after meeting them electronically; it always smooths the introduction for a face to face meeting.

Respondent #23

I’ve reached out to and/or interacted with professionals who I wouldn’t have known about or have been able to contact otherwise.

Respondent #9

Through the elimination of traditional barriers to travel and location, the use of Twitter allows for outreach and network building to be carried out more efficiently than conventional means. This inclusion of networked professionals outside the original user’s geographical area also increases diversity of ideas and experiences shared.

#### Continuing Education

The majority of public health professionals are required to have formal continuing education units (CEUs) to ensure that they are up to date with current practices in their field. The following open responses show that public health professionals engage in an informal type of CEU when engaging with other public professionals:

It is interesting to see their perspective on different issues and it challenges me to look at things from a different angle, with lenses that I may not have. It has certainly challenged my perspective on a number of issues.

Respondent #18

To keep up to date with recent developments. Get an impression of the broader public/Twitter users’ feeling on issues.

Respondent #26

It provides learning opportunities in terms of expanding my ideas, knowledge and perspectives by reading others’ ideas.

Respondent #39

This type of informal CEU is important to ensure that public health professionals are staying up to date on the latest news and research in the field, especially in a time with diminishing funding for formal CEU.

#### Professional Gain

A large part of professional development is networking to make connections and strengthening one’s profile as a public health professional. More and more professionals are blogging, using coordinated efforts on sites such as LinkedIn, YouTube, Pinterest, and so on, to create their own brand as an expert in a specific field or topic. The following open responses show that individuals specifically use Twitter as a way to increase their career profile:

I originally started using Twitter in 2010 to promote my blog, Pop Health. Twitter led to many great connections and opportunities (eg, being asked to guest blog on other public health sites).

Respondent #4

Learning, networking, sharing knowledge, and gaining a bigger profile in the PH (Public Health) community.

Respondent #15

Twitter provides a unique platform for public health professionals to put themselves, and their work, out into a large portion of the public health community all at once, while also quantifying their reach through followers and analytics. This use of Twitter circumvents traditional methods of professional gain often occurring at conferences, other professional gatherings, and through formal writing.

#### Communication

Another significant aspect of professional development is communication, which allows public health professionals to exchange ideas and to collaborate efficiently among the various fields in public health.

However, Twitter allows me to converse with colleagues all day. We discuss current events, professional development opportunities, and I often ask my followers for specific resources/references relevant to a paper or project I am working on. I get lots of responses.

Respondent #25

Twitter has made it easier to tune into conversations taking place near and far and discover individuals and organizations with similar interests. Twitter has made my tasks easier by making these interactions possible and accessible in a public forum.

Respondent #19

Open responses show Twitter is being used to communicate ideas and collaborate while eliminating time and response barriers usually experienced through email and other forms of communication. More specifically, as seen in these examples, many public health professionals are using Twitter as a means for conducting informal research and crowdsourcing ideas for work or branding. Furthermore, Twitter not only allows users to search for those working in similar subfields, it also allows them to follow whoever they want, thus allowing for a custom interdisciplinary approach to their feed and connections.

## Discussion

### Principal Findings

Along with being a social media outlet to connect to peers, Twitter has become a digital platform for public health professionals to enhance their professional development in a time of continual budget cuts and lack of funding. Whereas many public health workers are still receiving formal professional development to satisfy continuing education credits or certification needs, budget cuts have often resulted in lower staffing, which does not allow for as much informal learning that used to occur more naturally among colleagues in a work location. Twitter specifically eliminates geographical and locational boundaries; it provides informal continuing education, opportunities for growing career profiles, and a space to converse and collaborate with other public health professionals, often by connecting those in the same field or in other subspecialties of public health through avatar biographies and hashtags. Furthermore, Twitter is often an integral information conduit for many followers, as new notifications are often alerted to them through their electronic devices in real time.

A consistent use of the words “connect” and “connected” was found in the open-response section of the survey. Users also characterize the sharing of information in an outward direction, with many respondents discussing building their “brand,” blogs, websites, and so on, as well as an inward direction, where it is obvious that many of these public health workers are using Twitter as a major source of obtaining news and current events, often to gain different perspectives. One respondent’s claim of seeking “Twitter users’ feelings on issues” shows that users are not only seeking information and current events through the SNS but also allowing the collective voice help shape how they feel about certain issues. The real-time global expanse of Twitter also appeals to many in public health as they seek the perspective of witnessing a potential health crisis in real time from those “on the ground.” To see an edited news package on, perhaps, an Ebola outbreak is different than reading how people are dealing with the issue in the moment, at the center of the outbreak. Public health workers often compromise our first line of defense for a health emergency; therefore, using Twitter allows workers to see a health crisis from a new perspective. Twitter can provide a timeline of an event and allows users to read the collective voice of the response, often providing opportunities for learning what the most critical issues are and observing how those present there are trying to coordinate services.

Through the elimination of traditional boundaries, Twitter encourages public health professionals to be more creative in their work and research, as they can receive instant feedback and new ideas from public health professionals around the globe through informal interdisciplinary teamwork. Interdisciplinary teamwork among public health professionals could promote better health outcomes and more successful behavior change interventions as interdisciplinary teamwork in medicine has been shown to improve overall health outcomes and patient quality of care [15]. This study reflects the specific areas in which public health workers are using Twitter and how it can have value for their work and professional development. This work, however, is often done in their free time, or on their personal devices, as many local health departments and organizations block SNS through their Internet filters. This study should further the discussion on the value of removing these blocks to allow public health professionals to truly connect and collaborate with others working in the field of public health, or even those in the community they serve.

### Limitations

This study’s sample comprised active Twitter users, making it likelier that their feedback would overall be more positive. The aim, however, focused on how Twitter could potentially be beneficial for public health professionals, and an engaged group of users was critical in acquiring this information. The second limitation was the small sample size. Although only 49 of the 200 participants were recruited, this study was exploratory and did not seek to discover trends emerging among all public health professionals. The small sample size limits our study’s generalizability. Looking at the research of how public health organizations are using Twitter, however, parallels how individual public health professional use Twitter, making findings more generalizable.

### Conclusions

Overall, Twitter provides a unique platform for professional development, enhancing the work and research being conducted in the field. This study shows how public health professionals self-reported the potential benefits of using Twitter, whereas future research can focus on content analysis of how they are actually using the tool.

## Abbreviations

APHA: American Public Health Association
CEU: continuing education units
MPH: Master’s in Public Health
SD: standard deviation
SNS: social networking site
SPSS: Statistical Package for the Social Sciences
